# Validation and results of a novel survey assessing decisional balance for a whole food plant-based diet among US adults

**DOI:** 10.3389/fnut.2022.958611

**Published:** 2022-09-29

**Authors:** Christine E. S. Jovanovic, Faiza Kalam, Frank Granata, Angela F. Pfammatter, Bonnie Spring

**Affiliations:** ^1^Department of Minority Health Research, College of Medicine, University of Illinois at Chicago, Chicago, IL, United States; ^2^Department of Preventive Medicine, Feinberg School of Medicine, Northwestern University, Evanston, IL, United States

**Keywords:** whole food plant-based diet, nutrition behavior, decisional balance, confirmatory factor analysis, psychometric properties, consumption pattern

## Abstract

**Importance:**

Consuming a whole food plant-based diet (WFPBD) is a promising, low-risk strategy for reducing risk of prevalent chronic disease and certain cancers, with synergistic benefits for climate and environment. However, few US adults report consuming a WFPBD. Understanding the reasons for this inconsistency is important for developing and implementing interventions for promoting a WFPBD. However, no research to elucidate decisional balance driving current consumption patterns in the US exists.

**Objective:**

This research aims to validate an online survey to assess decisional balance for the consumption of a WFPBD, describe attitudes and beliefs toward adopting a WFPBD, and evaluate socio-demographic differences in decisional balance for consuming a WFPBD among a convenience sample of US adults.

**Design:**

Online cross-sectional data collection followed by confirmatory factor analysis (CFA), validation of internal consistency, and examination of invariance across socio-demographic variables. Sensitivity analysis of full vs. truncated survey to predict self-reported dietary patterns and consumption behaviors were evaluated. Results of the survey and significant differences by socio-demographics were assessed.

**Setting:**

Online survey based on previous research, created *via* Qualtrics, and administered through MTurk.

**Participants:**

A total of 412 US adults, majority female (66%), White (75%), 30–60 years old (54%), ≥ Bachelor’s degree (85%), and earning ≥ $45K (68%).

**Main outcomes and measures:**

Factor loadings, covariance of survey items, associations with self-reported dietary pattern and consumption measures, and differences in pros, cons, and decisional balance across socio-demographic variables.

**Results:**

CFA reduced the survey from 49 to 12 items and demonstrated invariance across socio-demographic variables. Pros and cons varied inversely and significantly (cov = –0.59), as expected. Cronbach’s α ’s for subscales in the final, reduced model were high (>0.80). Pros, cons, and decisional balance in both the full and the reduced model were significantly (*p* < 0.05) associated with self-reported dietary pattern and consumption.

**Conclusion and relevance:**

Our analyses indicate the WFPBD Survey is a parsimonious and psychometrically sound instrument for evaluation of decisional balance to consume a WFPBD diet among our sample of US adults. These results may be instrumental for development and deployment of interventions intended to promote consumption of a WFPBD in the US.

## Background

Diet is a critical component of human health (1–3) and is strongly implicated in the incidence of several chronic diseases, including cardiovascular disease (CVD), coronary artery disease (CAD), overweight and obesity, and type 2 diabetes (T2D), as well as certain cancers (4–10). Conversely, a whole food plant-based diet (WFPBD), defined as a pattern of consumption emphasizing minimally processed fruits, vegetables, legumes, nuts, seeds, and whole grains while minimizing meat, eggs, and dairy (11), has been shown to have numerous positive effects on overall human health (12–17), reducing risk for metabolic syndrome (18), obesity (19, 20), CVD (21, 22), T2D (23–25), and several diet-related cancers (26, 27). Additionally, increasing consumption of plant-based foods and decreasing consumption of meat has synergistic environmental impacts for reducing greenhouse gas emissions, decreasing land and water use, and protecting biodiversity (28–32). Widespread adoption and normalization of a WFPBD may simultaneously reduce the incidence of chronic disease and cancer while improving the environmental footprint of diet (31, 33).

Despite these potentially substantial benefits to human health and the environment, the number of people in the U.S. who report consuming a WFPBD remains very low (∼5%) relative to those who consume other, more traditional Western diets (34). In fact, less than 15% of adults in the US meet recommended levels of fruit and vegetable (FV) consumption (35). In 2017, per capita meat consumption in the US was 217 pounds, or about 3 times the global average (36), despite the increasing evidence in favor of WFPBD in promoting human and environmental health. Understanding the reasons for this disconnect is the first step in designing policies and interventions that may be effective in increasing uptake of a WFPBD in the US.

Research in Australia, Scotland, Portugal, and the Netherlands has revealed that attitudes and beliefs toward a WFPBD exhibit variation within and among sociodemographic and ethnic groups, and these differences vary among countries and over time (11, 30, 37, 38). For example, consumers in Europe reported significant differences in attitudes toward beef and pork, and these differences varied significantly across countries (39). In the US, the ADAPT study used a single multiple-choice question to find significantly more plant-based diet followers (including vegans, vegetarians, and pescatarians) compared to omnivores identified helping the environment and animal welfare as their top motivations (40). Other research in the US has posited philosophical reasons for consuming a plant-based diet, such as motivations, aversions, and constraints (41). However, there is a paucity of validated tools for assessing psychosocial constructs relevant for understanding current decisional balance driving consumption of a WFPBD in the US. Thus, we propose a pragmatic and theory-driven approach to developing tools and evidence to guide the design and implementation of interventions and policies intended to increase consumption of a WFPBD. This research will address this goal by achieving the following aims:

(1) Validate an online survey tool to assess attitudes and beliefs related to the consumption of a WFPBD among a convenience sample of 412 adults in the US,
(2) Describe attitudes and beliefs toward adopting a WFPBD among a sample of US adults, and
(3) Evaluate socio-demographic differences in attitudes and beliefs toward WFPBD among a sample of US adults.

## Materials and methods

### Survey development

The WFPBD survey was organized around the theory of decisional balance (pros vs. cons), posited by Janis and Mann (42), elaborated by Prochaska in the Transtheoretical Model (43–45), and found to be successful in predicting dietary behaviors (43–45). Questions were adapted from previous surveys administered in US and non-US populations (11, 37, 38, 46–48). Forty-nine survey questions adapted from Lea, Crawford, and Wolsey’s survey of 415 Australian adults were formatted as 5-item Likert scales, with responses: strongly disagree/disagree/not sure/agree/strongly agree. Items were selected to represent a two-factor model (pros and cons) with 23 items assessing cons of a WFPBD and 26 items assessing pros. To provide a theoretical and organizational framework, these 49 items were grouped to correspond to seven psychosocial constructs from Social Cognitive Theory (SCT) for each factor: social support, instrumental support, self-efficacy, perceived health impacts, taste preferences, knowledge, and attitudes toward animals. SCT constructs have been shown to be significantly associated with dietary behavior change in a variety of populations (49, 50). In addition, the survey contained demographic questions (5 items), dietary questions (6 items) adapted from the American Heart Association’s Rapid Diet Screener Tool for Clinicians (51) and 3 questions assessing motivational willingness to reduce meat consumption sourced from a previously validated survey (37), for a total of 77 questions (Supplementary Appendix A). The survey was administered using Qualtrics (Qualtrics©, Provo, UT, US) between August 30 and 31, 2021.

### Participant screening and recruitment

Participants were screened and recruited *via* Amazon’s Mechanical Turk (MTurk), an online platform that has been shown to offer a larger and more representative sample pool than traditional forms of convenience sampling can achieve (52–55). An initial screening survey was administered based on the study’s inclusion criteria: 18 years or older, fluent in English, and a resident of the US. Those who met the inclusion criteria were then directed to the consent document and asked to participate. If consent was given, the participant was then presented with the survey, and upon completion (as validated by a unique completion code generated once the survey was completed), the participant received $10 in compensation *via* the MTurk platform. Approval for this study was granted by the Institutional Review Board of Northwestern University (IRB ID: STU0054672).

### Analyses

Confirmatory Factor Analysis (CFA) was employed to validate the survey, and to determine if a more parsimonious tool would be as effective in capturing subject’s attitudes and beliefs toward a WFPBD, thus reducing survey burden. To achieve this, a CFA model was fitted using pros and cons of a WFPBD as latent variables that explained variance in relevant survey items. Because latent variable data were ordinal and not multivariate normal, ordinal logistic regression within a generalized structural equation model was used. For each factor (pros and cons), self-efficacy was used as the referent construct. Following the first CFA with all 49 items, those items with factor loadings < 0.80 were removed from the model. Then, modification indices were examined and item pairs with χ^2^ > 3.84 were assessed, and the variable with the lower factor loading was removed from the model. The same process was followed a second time to arrive at the final model. Cronbach’s α ’s were used to assess internal consistency. Model fit statistics were generated (AIC, BIC), and Average Variance Explained (AVE) and Discriminant Value (DV) calculated to provide measures of convergent and divergent validity, respectively. Invariance testing of the final model across sex, age, race/ethnicity, education, and income was performed to assess whether factor loadings varied significantly by demographic category.

Following CFA, pros and cons were evaluated following procedures originated by Janis and Mann (42) and utilized across a number of health behaviors (smoking, physical activity, diet) over several decades (43, 44, 56–59). First, pros and cons were calculated as the summed average grouped by factor (i.e., pros and cons), and decisional balance was calculated as the difference between pros and cons (pros minus cons). In addition, a categorical variable for each construct was generated, with 0 = strongly disagree/ disagree/ not sure and 1 = agree/ strongly agree. We compared the reduced WFPBD Survey to the original, using linear regression to assess whether the pros, cons, and decisional balance extracted from the shorter survey (independent variables) were able to predict self-reported consumption behaviors (i.e., self-reported daily servings of fruits and vegetables, and of meats, eggs, and dairy) (51) (dependent variables) at least as well as the original, adjusted for relevant demographic variables (age, income, education, and race/ethnicity). Finally, pros, cons, and decisional balance using the reduced, final model of the WFPBD Survey were evaluated, and significant differences, alone and across relevant demographic variables, were assessed *via* chi-square tests. Significance was assessed as a *p*-value < 0.05. All analyses were conducted using STATA version 16.1 (StataCorp (60)).

## Results

Our sample was primarily White (75%), 30–60 years old (54%), had a Bachelor’s degree or higher (85%), and earned $45K or more (68%). Close to one third of participants self-reported consuming no special diet and one-third self-reported consuming a vegetarian diet (no meat, includes dairy), with remainder evenly split (∼14%) between vegan (no animal products), pesco-vegetarian (vegetarian plus fish), and flexitarian (mostly vegetarian with minimal animal products) dietary patterns (Table 1).

**TABLE 1:** Demographic characteristics of the WFPBD survey sample.

	Total sample (*N* = 412)
**Sex**	***n* (%)**
Male	272 (66.02)
Female	139 (33.74)
Non-binary	1 (0.24)
**AGE**	
< 30 years	178 (43.10)
Between 30 and 60 years	222 (53.75)
> 60 years	13 (3.15)
**Ethnicity**	
African American/Black	48 (11.65)
Latino	25 (6.07)
White	309 (75.00)
Asian	25 (6.07)
Other	5 (1.21)
**Education**	
Less than Bachelor’s	63 (15.29)
Bachelor’s or higher	349 (84.71)
INCOME	
< $45,000/year	133 (32.28)
≥ $45,000/year	279 (67.72)
**Diet pattern**	
No special diet	113 (27.43)
Vegan	59 (14.32)
Vegetarian	121 (29.37)
Pesco-vegetarian	59 (14.32)
Flexitarian	60 (14.56)

### Survey validation

**FIGURE 1:**
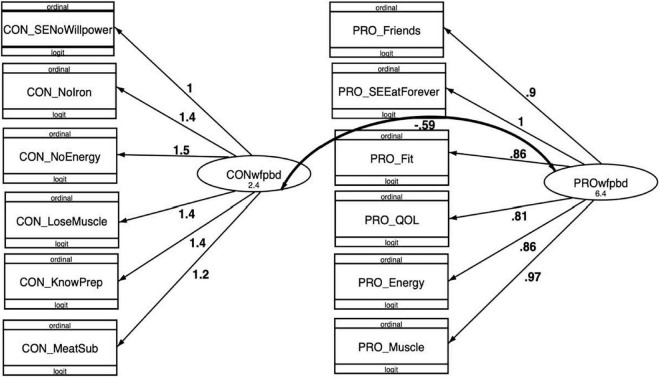
Final (12-item) generalized logit confirmatory factor analysis (CFA) model for WFPBD Survey, with factor loadings and covariances.

Following initial CFA, the model was iteratively reduced based on cut-points of 0.80 for factor loadings and 3.84 for modification indices, which yielded a final model with 6 items each for pros and cons of a WFPBD. In the full model (Model 1), highest factor loading among WFPBD pros was 0.97 for Q67 (“It helps me build or maintain muscle.”) and lowest was 0.81 for Q64 (“Generally, eating a WFPBD gives me a better quality of life.”), while for cons items the highest factor loading was 1.46 for Q32 (“I wouldn’t get enough energy or strength.”), and lowest was 1.25 for Q36 (“I don’t know what to eat instead of meat.”). In all models, all factor loadings were significant. Cronbach’s α ’s for both pros and cons subscales in the final, reduced model (Model 2) were high (> 0.80) (Table 2). Covariance between pros and cons of a WFPBD was –0.59 (*p* < 0.05), so that these latent variables are significantly and inversely related, as hypothesized (Figure 1).

**TABLE 2:** Factor loadings (SE), *p*-values, 95% CI’s, and R-squared statistics for the final WFPBD following confirmatory factor analysis.

WFPBD survey item	Factor loading (SE)	*P*-value	95% CI	*R* ^2^
**PROs subscale: Cronbach’s** α **= 0.88**				
Q47. My friends and family think eating a WFPBD is best.	0.90 (0.11)	<0.001	0.69, 1.12	0.52
Q53. I am confident I can eat a plant-based diet forever.	1.00 (Constrained)	NA	NA	0.58
Q63. A WFPBD is part of being fit.	0.86 (0.12)	<0.001	0.63, 1.09	0.55
Q64. Generally, eating a WFPBD helps me have a better quality of life.	0.81 (0.11)	<0.001	0.60, 1.02	0.56
Q65. I have plenty of energy on a WFPBD.	0.86 (0.12)	<0.001	0.63, 1.09	0.55
Q67. It helps me build or maintain muscle.	0.97 (0.12)	<0.001	0.74, 1.20	0.55
**CONs subscale: Cronbach’s** α **= 0.86**				
Q26. I do not feel confident that I have enough willpower to eat a plant-based diet.	1.00 (Constrained)	NA	NA	0.41
Q30. There is not enough iron in plant foods.	1.42 (0.19)	<0.001	1.05, 1.78	0.52
Q32. I wouldn’t get enough energy or strength.	1.46 (0.20)	<0.001	1.06, 1.85	0.57
Q35. I would lose muscle.	1.40 (0.19)	<0.001	1.03, 1.76	0.51
Q42. I don’t know how to prepare plant-based meals.	1.38 (0.17)	<0.001	1.04, 1.72	0.54
Q43. I don’t know what to eat instead of meat.	1.25 (0.16)	<0.001	0.94, 1.56	0.50

Model fit was assessed using AIC and BIC. With each iteration of the model, AIC and BIC decreased, indicating the model fit increased as factor loadings were optimized, cross loadings minimized, and items reduced (Table 3: Model Fit Statistics for CFA iterations of the WGPBD Survey). For the final model, convergent validity was explored using AVE and discriminant validity was assessed *via* the DV. A model is generally regarded as having acceptable convergent validity if the AVE is at least 0.50 and good convergent validity at an AVE above 0.70. Our final model resulted in an AVE of 0.61 for pros of a WFPBD and 0.57 for cons, such that the model has acceptable convergent validity. DV’s for the latent variables were both above recommended cut point of 0.70 (pros DV = 0.90, cons DV = 0.85), allowing us to conclude that the final model also displayed good divergent validity (61). Invariance testing was performed to assess whether factor loadings varied significantly across relevant demographic characteristics: age, sex, race/ethnicity, education, and income. Across all variables, the final model was invariant, with no significant differences among factor loadings for either pros or cons, indicating that the survey was consistent in measuring pros and cons across age, sex, race/ethnicity, education, and income.

**TABLE 3:** Model fit statistics for confirmatory factor analysis iterations of the Whole food Plant-Based Diet Survey.

	Model 1	Model 2	Model 3
Number of items	49	21	12
Log likelihood	–30,533.37	–23,924.99	–7935.58
AIC	61,678.73	48,321.92	16,033.15
BIC	62,909.16	49,270.95	16,358.36

To compare the original WFPBD Survey (Model 1, 49 items) to the final version (Model 3, 12 items), we fit linear regressions to assess whether the pros, cons, and decisional balance extracted from the final survey (independent variables) were able to predict self-reported consumption behaviors (i.e., self-reported daily servings of fruits and vegetables, and of meats, eggs, and dairy) (dependent variables) at least as well as the original, adjusted for relevant demographic variables (age, sex, race/ethnicity, education, and income). A similar pattern was found for both surveys, with significant β ’s for predicting fruit and vegetable consumption and meat, egg, and dairy consumption, with the notable exception of pros variables to predict fruit and vegetable consumption for both the original (Model 1) and the final reduced (Model 3) WFPBD Survey (Table 4).

**TABLE 4:** Pros, cons, and decisional balance (IVs) and self-reported consumption outcomes (fruit and vegetables, and meat, eggs, and dairy) (DVs) using logistic regression adjusted for age, sex, income, race/ethnicity, and education.

	Model 1 (49 items)		Model 3 (12 items)	
	β (SE)	*P-*value	β (SE)	*P-*value
**Fruits and vegetables**				
Pros	0.10 (0.14)	*p* = 0.47	0.57 (0.11)	*p* = 0.60
Cons	–0.71 (0.10)	*p* < 0.001	–0.56 (0.08)	*p* < 0.001
Decisional balance	–0.03 (0.01)	*p* < 0.001	0.38 (0.01)	*p* < 0.001
**Meat, eggs, and dairy**				
Pros	–0.85 (0.22)	*p* < 0.001	–0.63 (0.17)	*p* < 0.001
Cons	0.83 (0.17)	*p* < 0.001	0.62 (0.13)	*p* < 0.001
Decisional balance	0.02 (0.01)	*p* < 0.001	–0.53 (0.01)	*p* < 0.001

## Survey results

In the final model (Model 3), mean value for pros (3.76) was slightly higher than for cons (3.03), and both were significantly associated with dietary pattern (*p* < 0.001 for both). Logistic regressions revealed that the crude and adjusted models both show that decisional balance is a significant predictor (*p* < 0.001) of dietary pattern. In the adjusted model, compared to those who reported consuming “No special diet,” a one-unit increase in decisional balance is associated with a 2.14 times increased likelihood of reporting a vegan dietary pattern (no animal products) and a 1.55 times increased likelihood of reporting a flexitarian diet (mostly vegetarian with small amounts of meat and fish) (62). Overall effect of education is statistically significant in predicting the relationship between decisional balance and diet pattern (χ^2^ = 45.54, *p* < 0.001). However, no significant interactions between education and decisional balance were found.

**FIGURE 2:**
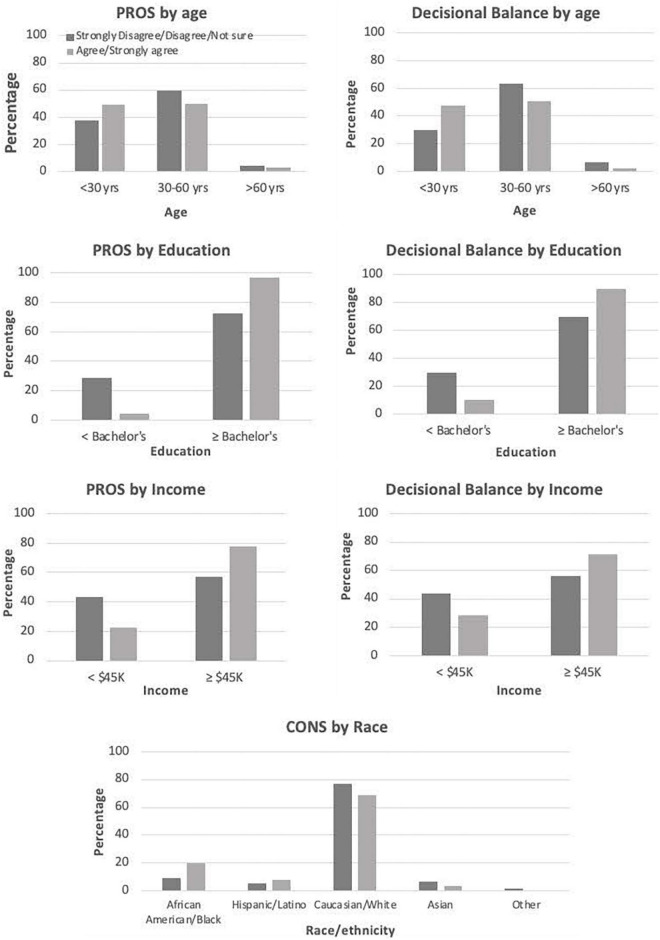
Significant (*p* < 0.05) differences in pros, cons, and decisional balance by demographic variables.

Evaluating the final model using χ^2^ tests, significant differences (*p* < 0.05) for pros, cons, and decisional balance were found across demographic variables. For pros and decisional balance, significant differences by age, education, and income were found, while cons varied significantly only by race/ethnicity (Figure 2). For age, those less than 30 years old had higher pros, while those between 30 and 60 years had higher cons. Participants older than 60 were ambivalent, exhibiting low levels of both pros and cons. Education also varied similarly for pros and decisional balance, with those with at least a Bachelor’s degree expressing higher agreement with pros and positive decisional balance. Also of note were the relatively higher cons and negative decisional balance for those with less than a Bachelor’s degree. Income followed the same pattern, with higher pros for those earning $45K or higher, and higher cons for those making less than $45K.

## Discussion

The CFA of the WFPBD Survey resulted in a more parsimonious version that exhibited high factor loadings and strong validity, reducing the number of items from 49 to 12. This smaller number of items reduces survey burden and improves the utility of the WFPBD survey for inclusion in other survey instruments. Invariance testing revealed no significant differences in factor loadings across demographic variables, suggesting that the final WFPBD Survey is valid across a variety of demographics. Cronbach’s alphas were above the 0.70 cutoff to indicate good internal consistency, and below 0.90, indicating minimal redundancy among the questions (63). Pros and cons of a WFPBD covaried inversely, as expected (β = –0.69, *p* = 0.03), demonstrating that pros increase as cons decrease, and vice versa. Both convergent validity (as expressed by the AVE above 0.50 for both pros and cons) and divergent validity (DV > 0.50 for pros and cons) were within acceptable ranges, indicating that the final survey items measured latent constructs as hypothesized. Further analysis of the final model revealed that the theoretical framework of pros, cons, and decisional balance was associated, as expected, with self-reported dietary pattern, providing further evidence that the reduced WFPBD Survey was able to measure the latent variables as intended. Our comparison of the original vs. reduced WFPBD Survey for predicting self-reported consumption of fruit and vegetables and meat, eggs, and dairy provides evidence that the reduced survey can elucidate significant predictors of these dietary behavior targets. Thus, by several important metrics, the WFPBD Survey demonstrates stable and high factor loadings, good internal consistency, acceptable levels of convergent and discriminant validity, and factor invariance, providing evidence that the reduced survey successfully measured the intended constructs, namely the pros, cons, and decisional balance for consuming a WFPBD in a sample of US adults. Additionally, our findings that pros and cons successfully predicted self-reported dietary intakes suggests that these psychosocial constructs are important for driving dietary choices, and, thus, accessible targets for interventions that seek to encourage consumption of a WFPBD.

The WFPBD Survey was able to detect significant differences among our sample, with decisional balance of pros and cons tracking with dietary pattern, and revealing important and significant variations across income, education, and race/ethnicity. For example, cons were significantly higher for Hispanic and African American participants, suggesting that barriers to consuming a WFPBD may be most important in these populations, as compared to pros. Being less than 60 years old, making at least $45K annually, and having at least a Bachelor’s degree were all significantly associated with higher pros and lower cons for a WFPBD. This aligns with other research, such as Lea et al.’s (11) survey assessing perceptions of the benefits and barriers to eating a plant-based diet among 415 Australian adults. In this study, significant differences in perceived benefits and barriers were detected by sex, age, and education, similar to our results (11). Other research has found consumer attitudes and behaviors vary by country and have changed over time, even within the same demographics and countries. For instance, research in both Finland (64, 65) and Europe (39) has discovered shifts in consumer concerns around consumption of meat, from a more safety-focused paradigm to one that prioritizes health implications. Similarly, Fresán et al. (66) found motivations for consuming a plant-based diet to vary between California (health, social norms, religion) and Copenhagen (animal welfare, health, environment) (66). While these studies provide some insight into the reasons consumers may or may not chose to consume a WFPBD, these heterogenous results are attributable to culturally specific and dynamic processes and cannot be assumed to translate to adults in the US currently. Research in the US has found important differences in motivations between plant-based consumers and omnivores (40, 41), but did not validate the surveys used in these studies. This highlights the literature gap which this study aims to fill by providing a validated instrument suitable for US adults, as well as results from this convenience sample. These results may be instrumental for development and deployment of interventions intended to promote consumption of a WFPBD in US adults. In particular, the failure of pros to predict self-reported consumption of fruits and vegetables along with significant associations for decisional balance and cons, suggests that addressing cons in future interventions is the more effective approach to promoting consumption of a WFPBD. Additionally, our findings of significant differences in cons across race/ethnicity suggests that tailoring interventions with particular attention to racial/ethnic differences is important, especially for African American and Hispanic populations.

Limitations of this study include the lack of a test-retest reliability measure, since the survey was taken only once. In addition, all measures of both dietary pattern and consumption were self-reported, and therefore potentially subject to a number of biases, including imprecise recall and social desirability bias. Finally, this sample has a higher percentage of White participants (61.6% for the US vs. 75% for this study) and fewer Hispanic participants (18.7% for the US vs. 6.07% for this study) than the US in 2020 (67). Our sample was also more educated than average (84.71% for this study vs. 37.5% in the US with a Bachelor’s degree or higher (68)) and more affluent (67.72% for this study vs. 53.74% in the US earning $45K or more (69)). Given the significant differences in pros, cons, and decisional balance we found across age, income, and education, the differences between this study’s population and the US population are relevant for judging the generalizability of our results.

While our results elucidate several important and significant variations that may be useful for tailoring pro-WFPBD interventions to adult US participants by age, education, and income, further research is needed to assess whether these differences are consistent in other groups, such as Hispanic audiences, immigrant populations, or less affluent people. These differences were not detected in our relatively non-diverse sample. However, the validation measures we report here may be useful in achieving this goal by providing a psychometrically sound instrument with which to assess these psychosocial constructs in diverse populations. For example, a WFPBD has been shown to reduce levels of prostate-specific antigen in biopsy-confirmed indolent prostate cancer (70), which disproportionately affects African American men in US (71). The WFPBD Survey may be an important first step for understanding how to design and deploy an effective intervention to promote consumption of a WFPBD among African American men with indolent prostate cancer. The results from this study, as well as future uses of the WFPBD survey, provide evidence upon which we can build interventions to encourage a WFPBD for better health and environmental outcomes in the US.

## Data availability statement

The raw data supporting the conclusions of this article will be made available by the authors, without undue reservation.

## Ethics statement

The studies involving human participants were reviewed and approved by Institutional Review Board of Northwestern University (IRB ID: STU0054672). The patients/participants provided their written informed consent to participate in this study.

## Author contributions

CJ designed the study, conducted primary data collection, performed data analysis, created tables, and served as primary author. FK assisted in writing the study proposal, created figures, and collaborated on writing the manuscript. FG assisted in writing the study proposal and collaborated on writing the manuscript. AP assisted in developing the survey and edited the manuscript. BS assisted in developing the study design, edited the manuscript, and provided funding. All authors contributed to the article and approved the submitted version.
