# Supplementary material for: Validation and results of a novel survey assessing decisional balance for a whole food plant-based diet among US adults
Source: Front Nutr. 2022 Sep 29;9:958611. doi: 10.3389/fnut.2022.958611 (PMC9557160; doi:10.3389/fnut.2022.958611)
Supplement: Supplementary file 1 [file Image_1.pdf]

WFPBD Survey

Attitudes and beliefs towards a whole food plant-based diet

Christine Jovanovic, PhD MPH

Screener Questionnaire

QUESTION		RESPONSE
**#**		
**1**	Are you 18 years old or older?	☐ Yes<br>☐ No
**2**	Can you read and write English fluently?	☐ Yes<br>☐ No
**3**	Do you reside in the United States?	☐ Yes<br>☐ No

WFPBD Survey

The questions for this survey have been adapted from Leah et al. (2006) and Evans (2020).

QUESTION		RESPONSE
The following questions ask about your diet, and your attitudes and beliefs towards a whole food plant-based diet. <u>We will not share your answers with anyone outside the research team.</u> This survey is completely anonymous and confidential. Please answer the questions to the best of your ability by checking or circling <u>the answer that is best for you</u>. Thank you!		
**#**	**Demographics**	
**1**	What is your biological sex?	☐ Male<br>☐ Female<br>☐ Prefer not to answer
**2**	What is your age?	Text
**3**	What is your race/ethnicity?	☐ African American or Black<br>☐ Hispanic or Latino<br>☐ Caucasian or White<br>☐ Asian<br>☐ Other
**4**	What is the highest grade or level of school you completed or the highest degree you have received?	☐ No diploma<br>☐ High School<br>☐ Associate degree or technical college<br>☐ Bachelor’s degree<br>☐ Master’s degree<br>☐ PhD or equivalent degree
**5**	What was your <u>annual</u> household gross income for 2020?	☐ Under $25,000<br>☐ $25,001-$45,000<br>☐ $45,001-$65.000<br>☐ $65,001-$85.000<br>☐ Greater than $85,000
**#**	**Dietary Questions**	

**6**	Do you follow a special diet?	☐ Vegan (no animal products)<br>☐ Vegetarian (includes eggs and dairy)<br>☐ Pesco-vegetarian (includes fish, eggs, and dairy)<br>☐ Flexitarian (minimal meat, eggs, dairy)<br>☐ No special diet
**7**	What is the total amount of fruit you usually eat each day? (1/2 cup equals approximately 1 handful.)	☐ 0 cups<br>☐ ½ cup<br>☐ 1 cup<br>☐ 1 ½ cup<br>☐ 2 cups or more
**8**	What is the total amount of vegetables you usually eat each day? (1/2 cup equals approximately 1 handful.)	
**9**	What is the total amount of dairy you usually eat each day, (1/2 cup of milk or yogurt is equivalent to 1 ounce of cheese, etc.)	
**10**	What is the total amount of chicken, beef, pork, or fish you usually eat each day? (1/2 cup equals approximately 1 palm-sized portion, or 3 ounces.)	
**11**	On average, how many times per week do you eat meat? This includes all meat-based products (e.g., chicken, beef, pork, fish, etc.)	I eat meat…<br>☐ …for the majority of my meals (including breakfast, lunch and dinner)—more than 10 times per week<br>☐ …for most of my main meals—about 7-10 times per week<br>☐ …a few times per week—about 4-6 times per week<br>☐ …almost no meat—about 1-3 times per week<br>☐ I do not eat meat
**12**	What is the total amount of eggs you usually eat each day.	☐ 0 eggs<br>☐ 1 egg<br>☐ 2 eggs<br>☐ 3 eggs<br>☐ 4 eggs or more

Some people believe that eating a plant-based diet has specific challenges. How much, if at all, do these statements apply to you? Please choose one option. **Eating a plant-based diet would be or is *difficult* for me because…**

#	Cons (Barriers)	
**Social Support**		
**13**	I don't want people to think I'm weird or pretentious.	☐ Strongly disagree<br>☐ Disagree<br>☐ Not sure
**14**	My family/partner won't eat a plant-based diet.	
**15**	I am uncomfortable ordering "special" meals.	

**16**	I don’t want to ask my friends and/or family to cook plant-based meals when I visit.	☐ Agree<br>☐ Strongly agree
**17**	I am concerned I would have to give up my food heritage/culture.[1]	
**18**	It would mean conforming to the dominant culture.[1]	
**19**	I can afford to eat meat.	
**Instrumental Support**		
**20**	It is inconvenient.	☐ Strongly disagree<br>☐ Disagree<br>☐ Not sure<br>☐ Agree<br>☐ Strongly agree
**21**	The plant foods I would need aren't available where I shop or in the cafeteria or at my home	
**22**	It takes too long to prepare whole plant-based meals	
**23**	Plant-based meals or snacks are not available when I eat out.	
**24**	There is not enough choice when I eat out.	
**25**	It would be too expensive.	
**Self-Efficacy**		
**26**	I do not feel confident that I have enough willpower to eat a plant-based diet.	☐ Strongly disagree<br>☐ Disagree<br>☐ Not sure<br>☐ Agree<br>☐ Strongly agree
**27**	I do not feel confident that I can find plant-based foods when I eat out.	
**28**	I am not confident that I can buy plant-based foods at the grocery store.	
**29**	I am not confident that I know how to prepare plant-based meals.	
**Perceived Health Disadvantages/Harms**		
**30**	There is not enough iron in plant foods.	☐ Strongly disagree<br>☐ Disagree<br>☐ Not sure<br>☐ Agree<br>☐ Strongly agree
**31**	I would be worried about my health.	
**32**	I wouldn't get enough energy or strength.	
**33**	There is not enough protein in them.	
**34**	I would get indigestion, bloating, gas or flatulence.	
**35**	I would lose muscle.	
**Taste Preferences**		
**36**	It would not be tasty enough.	☐ Strongly disagree<br>☐ Disagree<br>☐ Not sure<br>☐ Agree<br>☐ Strongly agree
**37**	I would need to eat such a large quantity of plant foods.	
**38**	I don't want to change my eating habit or routine.	
**39**	I don't want to eat strange or unusual foods.	
**40**	It would not be filling enough.	
**41**	I would miss eating lots of ‘junk’ (e.g., sugary, salty) food.	
**Knowledge**		
**42**	I don't know how to prepare plant-based meals.	☐ Strongly disagree<br>☐ Disagree<br>☐ Not sure<br>☐ Agree<br>☐ Strongly agree
**43**	I don't know what to eat instead of meat.	
**44**	I need more information about plant-based diets.	
**Attitude Towards Animals**		
**45**	I think humans are meant to eat lots of meat.	☐ Strongly disagree<br>☐ Disagree<br>☐ Not sure
**46**	According to our position in the food chain, we have the right to eat meat.	

		☐ Agree<br>☐ Strongly agree

Some people believe that eating a plant-based diet has specific benefits. How much, if at all, do these statements apply to you? Please choose one answer. **I believe eating a plant-based diet could or does *help* me to…*or* I believe eating a plant-based diet can help…**

#	Pros (Benefits)	
**Social Support**		
**47**	My friends and family think eating a WFPBD is best.	☐ Strongly disagree<br>☐ Disagree<br>☐ Not sure<br>☐ Agree<br>☐ Strongly agree
**Instrumental Support**		
**48**	It is cheaper to eat a WFPBD.	☐ Strongly disagree<br>☐ Disagree<br>☐ Not sure<br>☐ Agree<br>☐ Strongly agree
**49**	It takes less time to prepare whole, plant-based foods.	
**50**	I have fewer food storage and spoilage problems.	
**51**	I can find good options to eat at restaurants.	
**Self-Efficacy**		
**52**	I would feel healthy and energetic eating a whole food plant-based diet.	☐ Strongly disagree<br>☐ Disagree<br>☐ Not sure<br>☐ Agree<br>☐ Strongly agree
**53**	I am confident I can eat a plant-based diet forever.	
**54**	I am confident I can cook meals using whole plant-based foods.	
**Perceived Health Advantages/Benefits**		
**55**	I would decrease my saturated fat intake.	☐ Strongly disagree<br>☐ Disagree<br>☐ Not sure<br>☐ Agree<br>☐ Strongly agree
**56**	I would eat more fiber.	
**57**	A WFPBD reduces the risk of disease in general (e.g., heart disease, cancer).	
**58**	A WFPBD is a more 'natural' diet.	
**59**	I would get lots of vitamins and minerals.	
**60**	It would help me stay healthy.	
**61**	It would help me control my weight.	
**62**	It would help improve my digestion.	
**63**	A WFPBD is part of being fit.	
**64**	Generally, eating a WFPBD helps me have a better quality of life.	
**65**	I have plenty of energy on a WFPBD.	
**66**	It lowers my chances of getting food poisoning.	
**67**	It helps me build or maintain muscle.	
**Taste Preferences**		
**68**	There are many delicious whole plant-based foods that I like.	☐ Strongly disagree<br>☐ Disagree<br>☐ Not sure<br>☐ Agree<br>☐ Strongly agree

Knowledge		
**60**	Eating a WFPBD helps the environment.	☐ Strongly disagree<br>☐ Disagree<br>☐ Not sure<br>☐ Agree<br>☐ Strongly agree
**70**	Plant-based foods Increase efficiency of food production.	
**71**	Increasing the consumption of WFPBD can decrease hunger in the world.	
**Attitude Towards Animals**		
**72**	It is important to support animal welfare/rights.	☐ Strongly disagree<br>☐ Disagree<br>☐ Not sure<br>☐ Agree<br>☐ Strongly agree
**73**	By eating meat I'm reminded of the death and suffering of animals.	
**Willingness to reduce meat consumption**		
**74**	I would be willing to reduce the amount of meat I eat for the sake of the environment.	☐ Strongly disagree<br>☐ Disagree<br>☐ Not sure<br>☐ Agree<br>☐ Strongly agree
**75**	I would be willing to reduce the amount of meat I eat if I could save money.	
**76**	I would be willing to reduce the amount of meat I eat for the sake of my health.	
